# Southern Elephant Seals Replenish Their Lipid Reserves at Different Rates According to Foraging Habitat

**DOI:** 10.1371/journal.pone.0166747

**Published:** 2016-11-30

**Authors:** Gaëtan Richard, Samantha L. Cox, Baptiste Picard, Jade Vacquié-Garcia, Christophe Guinet

**Affiliations:** CEBC-CNRS, Villiers-en-Bois, France; University of Saint Andrews, UNITED KINGDOM

## Abstract

Assessing energy gain and expenditure in free ranging marine predators is difficult. However, such measurements are critical if we are to understand how variation in foraging efficiency, and in turn individual body condition, is impacted by environmentally driven changes in prey abundance and/or accessibility. To investigate the influence of oceanographic habitat type on foraging efficiency, ten post-breeding female southern elephant seals *Mirounga leonina* (SES) were equipped and tracked with bio-loggers to give continuous information of prey catch attempts, body density and body activity. Variations in these indices of foraging efficiency were then compared between three different oceanographic habitats, delineated by the main frontal structures of the Southern Ocean. Results show that changes in body density are related not only to the number of previous prey catch attempts and to the body activity (at a 6 day lag), but also foraging habitat type. For example, despite a lower daily prey catch attempt rate, SESs foraging north of the sub-Antarctic front improve their body density at a higher rate than individuals foraging south of the sub-Antarctic and polar fronts, suggesting that they may forage on easier to catch and/or more energetically rich prey in this area. Our study highlights a need to understand the influence of habitat type on top predator foraging behaviour and efficiency when attempting a better comprehension of marine ecosystems.

## Introduction

Within an optimal foraging framework, net energy gain is the “currency” that individuals should aim to maximize [1,2]. It results from the balance between the energy expenditure associated with body maintenance and locomotion, and energy gain from prey consumption. However, assessing energy intake and overall energy expenditure, and subsequently net energy gain, is difficult to achieve in free ranging marine predators. This is predominantly because these animals are hard to observe and monitor at sea making direct measurement challenging. However, such information is critical if we are to understand how changes in prey abundance and/or accessibility impact an individual’s energy balance and subsequently its reproductive output.

In recent years, time depth recorders [3] in combination with high sampling rate accelerometers [4] have provided new insight toward the foraging efficiencies of a broad range of species. For example, it is now possible to both identify prey capture attempts (PCA; [5,6]) and estimate locomotion costs for female southern elephant seals *Mirounga leonina* (SES), the latter of which can be used as an indirect estimate of at-sea energy expenditure. This is especially appropriate to SESs, since basal metabolic rates are thought to be comparatively low, in part due to the efficiency of SES thermoregulation [7]. Day-to-day variation in body density (kg.m^-3^) can also be estimated, and used as a proxy of lipid/lean tissue ratio, by monitoring vertical speeds during either the drift phase of a resting/food processing dive (*i*.*e*. drift dive [8,9]), the descent phase of a foraging/travel dive, or the level of swimming effort (*i*.*e*. stroke rate and amplitude) during the ascent phase of a foraging/travel dive. Together these measurements can be used to address how changes in energy expenditure and foraging effort impact body condition and net energy gain.

Female SESs forage mainly on small fish from the myctophid family [10,11]. The species composition of communities of these prey, alongside their vertical distribution through the water column are known to vary across the main frontal structures of the Southern Ocean [12]. As such, prey accessibility and profitability for SESs may vary between oceanographic domains. Indeed, the foraging depths of SESs increase northwards from the Antarctic divergence to subtropical waters [13,14]. Subsequently, foraging costs and net energy gain may vary between oceanographic domains.

Following on from this, the purpose of this study was to assess how foraging efficiency may vary between contrasting oceanographic domains where the quantity and quality of prey may differ. We first examined quantitative differences in foraging activity between oceanographic domains by estimating variation in daily PCAs during SES foraging trips. Second, we assessed differences in foraging efficiency between oceanographic domains using improvement rates of SES body condition. Day-to-day changes in SES body density were used as a relative index of net energy gain which, whilst accounting for changes in PCAs and foraging costs, were compared between the three oceanographic domains within which the SESs forage. In this second analysis we also assessed whether changes in SES body density are linked to variation in PCAs, and therefore if PCAs are a good indicator for overall energy intake.

## Materials and Methods

### Ethics statement

Field permits were approved and authorized by the ethics committee of the Centre National de la Recherche Scientifique (CNRS) and the French Polar Institute (Institut Paul Emile Victor—IPEV- Comité de l'Environnement Polaire). All animals in this study were handled and cared for in accordance with the guidelines and recommendations of these committees (dirpol@ipev.fr). Manipulations of animals were conducted under the “authorization of experimentation for vertebrate species” permit of Christophe Guinet (n°7200). Both the Centre National de la Recherche Scientifique (CNRS) and the French Polar Institute (IPEV) approve this study.

### Study area and deployment of devices

The foraging efficiencies of ten post-breeding female SESs from the Kerguelen Islands (49°20’S, 70°20’E) were investigated during at-sea foraging trips between 2010 and 2014 (3 in each of 2010 & 2011, 1 in each of 2012 & 2013 and 2 in 2014). Females were equipped with (1) an Argos-GPS satellite relay tag, set to transmit Argos locations daily and archive GPS location data (Splash10-F, Wildlife Computer, USA), and (2) a time-depth recorder (TDR, Wildlife Computer, USA), logging pressure and temperature at 1 Hz, combined with a tri-axial accelerometer recording at 16 Hz (MK10-X, Wildlife Computer, USA) which was mounted to the head of an animal.

### Trajectories

**Fig 1:**
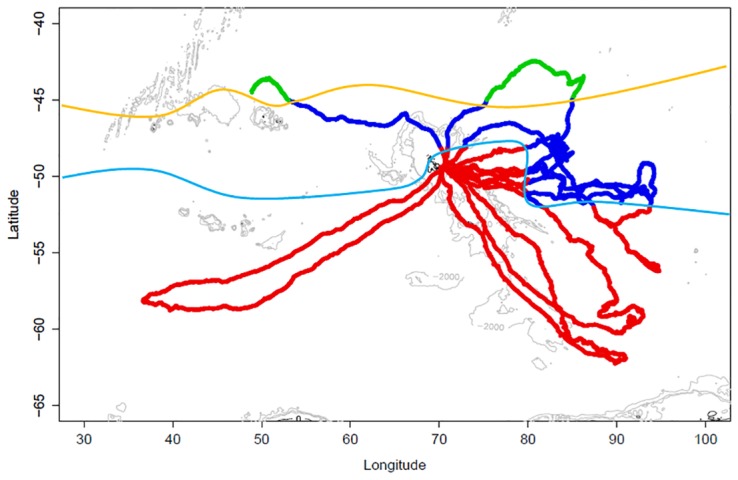
Distribution of the 3 oceanographic domains (habitat types) along each of the 10 trips performed by SESs: habitat #1 (green) is north of the sub-Antarctic front (yellow line), habitat #2 (blue) lies between the sub-Antarctic front and the polar front (cyan line) and habitat #3 (red) is south of the polar front. Frontal locations were defined using temperature and salinity measurements taken at 200m depth [26–28].

A GPS position was recorded during most SES surfacing events. A linear interpolation between the position fixes obtained from the GPS was used to estimate missing locations (Fig 1). Following this, a custom-written function in MATLAB (MathWorks, Natick, MA, USA) was used to estimate the horizontal distance covered during each dive (*i*.*e*. the distance between the GPS coordinates of the start and the end of each dive). A large horizontal distance between two surfacing GPS points suggests an animal is travelling through an area, whilst a small horizontal distance reflects a prolonged stay within an area. To investigate animal movements at a daily scale, this metric was also calculated using the GPS coordinates from the beginning and end of each 24 hour period along the animal’s trajectory.

### Proxies of foraging activity

#### Prey capture attempts

We assumed that overall energy intake could be estimated using PCAs, although it is noted that whilst this index provides an indication of prey encounter rates it does not provide any information on actual (successful) capture rates nor the size, species, and energy content of ingested prey. To identify PCAs, acceleration data were processed according to Viviant et al. [5] and Gallon et al. [6], using custom-written MATLAB code (available on request). First, the 3 (x, y and z) acceleration time series were filtered using a high pass filter with a critical frequency of 0.33Hz [5,6]. This process removed the gravity and swimming movement components of the acceleration signals, thus highlighting peaks in acceleration from rapid head movements. The 0.33 filter was defined visually from the power spectral densities of acceleration along the 3 axes. Secondly, standard deviations were calculated along a fixed 1s window for the 3 filtered acceleration axes individually. This process was then repeated along a 5s moving window over each previously calculated standard deviation time series, to highlight extreme standard deviations and consequently significant acceleration spurts. Kmeans clustering (from the statistic toolbox in MATLAB) was then used to isolate extreme events (indicative of rapid head movements) for each SES and along each axis independently. Only rapid head movement events which could be detected simultaneously on all of the 3 axes were considered as a true PCA. Continuous PCA events at 1Hz were considered as a single prey capture attempt. Events separated by periods longer than 1s for any given axes were considered as different PCAs. The number of PCAs performed by each individual SES was summarized for each day.

#### Body movements

A proxy of energy expenditure, overall dynamic body acceleration (ODBA), was estimated as the sum of the absolute dynamic acceleration from the three accelerometer axes [15–17]:

ODBA=|Ax|+|Ay|+|Az| (1)

where *A*_*x*,_ *A*_*y*_ and *A*_*z*_ are the dynamic accelerations (in *g*) of the x, y and z axes of the accelerometer respectively. Dynamic accelerations were obtained by subtracting the static acceleration, obtained by applying a low-pass filter of 0.2Hz [18], from the raw accelerations of each axis [15]. ODBA was estimated over a time scale of one day to approximate the daily expenditure associated with body movements.

### Rate of change in body density

SES body density was estimated following Richard *et al*. [19]. First, dives where SESs stopped actively swimming at the bottom (as indicated by a low vertical speed and null lateral acceleration, *i*.*e*. no stroking patterns [19,20]) were selected. During the drift phases of these resting dives, SESs are subject to move according to buoyancy and drag since no driving force is produced by the animals [9,21]. Thus, by measuring drift rates (*i*.*e*. vertical speeds) body density can be resolved using the net force equation derived from Newton’s Second Law [9,19,21,22]. Second, because on average drift dives embodied less than 2% of dives performed by the ten female SESs tracked, which would result in a low resolution of body density variation over the trip (*i*.*e*. seals did not perform drift dives every day [19]), we monitored absolute descent speeds (during the descent phase of a dive), which can be used as an estimation of relative variation in body density over the duration of a trip, without the need to estimate absolute body density values [19,23]. Finally, these two methods (absolute and relative measures of body density) were combined to provide continuous daily estimates of body density throughout each of the then SES’s tracks [19]. To calculate a rate of change in body density variation, we used a cubic smoothing spline function (*csaps*, Matlab). Several smoothing parameters were fitted and the best chosen based on visual observation (see S1 Fig). A parameter of 0.01, was selected allowing us to still capture general variation in body density variation whilst minimising noise. From these smoothed density curves, we then calculated the daily rate of the body density variation [22,24] over a moving-window of one day:

$$\text{Density variation rate}=\frac{\left(\rho \text{i}+1\;-\;\rho \text{i }\right)}{\rho \text{i }}$$ (2)

with *ρ* the body density, *i* the initial day of the moving windows.

A negative rate of change in body density indicates a decrease in body density, which can be considered as an improvement in body condition due to an increase in lipid content [9,19,21].

### Oceanographic domains

The horizontal and vertical distributions of most myctophid species are tightly linked to water temperature [12], which is thought to drive variation in SES foraging behaviour [13]). As such, following Guinet et al [13], foraging activity along each SES track was assigned one of three oceanographic domains (habitat types) according to the daily mean 200-m temperature [13,25–28], which was calculated from measurements from the TDR logger taken during dives occurring over each day of the trip. Domain habitats were separated by sharp changes in these temperatures, indicative of hydrological fronts [29]. Habitat 1 lies north of the sub-Antarctic front, habitat 2 between the sub-Antarctic front and the polar front, and habitat 3 south of the polar front (Fig 1).

### Statistical modelling

Statistical analyses were performed in R version 3.2.3 (R Development Core Team 2012). Linear mixed effects models (LMM), from the R package *nlme* [30], were used to determine (*i*) whether the number of PCAs performed over a day, which approximates a daily PCA rate and follows a normal distribution, is related to the distance travelled during that day and/or the oceanographic domain occupied (Eq 3, data are available in S1 File), and (*ii*) whether variation in the rate of change in body density (Eq 2) is related, at a time lag of six days, to the number of prey capture attempts, swimming activity (ODBA, Eq 1) and/or the oceanographic domain occupied (Eq 4, data are available in S2 File). A time lag of six days between the response and explanatory variables in the second analysis was selected as this is the period over which a true change in drift rate can be detected and separated from random noise [9]. This value also reflects those identified from literature investigating links between post breeding female SES area restricted search (ARS) behaviours and change in body density [31,32].

Daily PCA ~ daily horizontal distance + habitats (3)

Variation rate of body density(t) ~ PCA(t−6) + ODBA(t−6) + habitats(t−6) (4)

A random effect of individual was included in both analyses to allow for variation in feeding attempts and the rate of body density change between individuals. For both analyses, oceanographic domain was set as a categorical factor, with habitat 1 (north of the Sub-Antarctic Front) as the baseline category.

Residuals of both models followed a normal distribution allowing us to interpret the results.

## Results

### Foraging trips: trajectory and energetic balance

Across the 10 trips, the mean recording duration of the accelerometer loggers was 60 ±14 days (±standard deviation; range = 35 to 79 days). This comprised five complete trips (*i*.*e*. recording stopped when the seals were back in Kerguelen), and five incomplete trips (loggers ceased recording whilst SESs were still at-sea (Fig 1). All 10 SESs visited the oceanographic domain south of the polar front (habitat 3) since this is where Kerguelen Island is located. North of the sub-Antarctic front (habitat 1) was visited by 2 SESs, whilst 8 individuals visited the domain that encompassed the area between the sub-Antarctic front and the polar front (habitat 2). Further interpretation of the results should note the small number of samples obtained within habitat 1, and that these come from only two of the tracked individuals.

**Fig 2:**
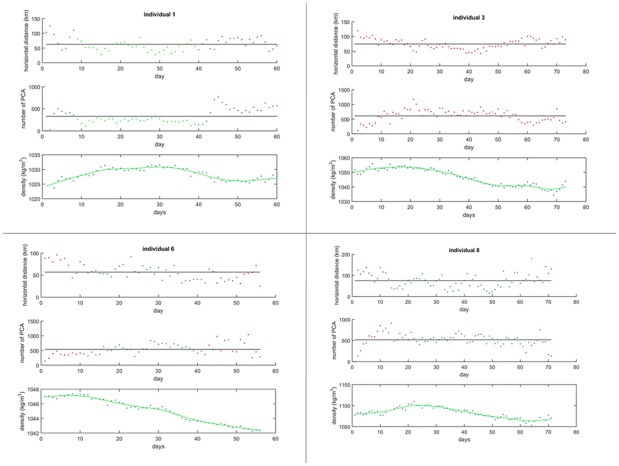
Daily horizontal distances, daily number of prey catch attempts and daily body densities across the trips of 4 SESs. Colours indicate the predominant oceanographic domain visited during each day: (1) habitat 1 in green, (2) habitat 2 in blue and (3) habitat 3 in red. See supporting information for plots for all individuals (see S2 Fig).

Compared to movements made during the middle of a trip (*i*.*e*. between 20 and 50 days: 59±23 km.day^-1^), individuals covered significantly higher horizontal distances at the beginning (*i*.*e*. 20 first days: 81±32 km.day^-1^, Student t-Test between distances during the beginning and the middle of trips: t = 6.9, p<0.001), and end (*i*.*e*. after 50 days, from only individuals for which a complete trip was recorded: 89±41 km.day^-1^, Student t- Test between distances during the end and the middle of trips: t = 6.6, p<0.001) of a trip, suggestive of fast and directed travel [33] (Fig 2).

Overall, seals performed 529 ±146 PCAs per day. Seals appeared to perform more PCAs during the middle of a trip (626±254 PCAs) when horizontal distances travelled were low, than during the beginning (440±212 PCAs) and the end (496±221 PCAs) of the trip (Fig 2). Using Student’s t-Tests, we confirmed that differences between the mean number of PCAs during the middle and the beginning (t = 8.5, p<0.001) and end of the trip (t = 5.1, p<0.001) were significant. However, this trend was not consistent across all oceanographic domains (Fig 2). Indeed, the 1^st^ individual performed fewer PCAs in habitat 1 (Fig 2 & S3 Fig) than the mean PCA number across its entire trip, even though the horizontal distance covered per day within this habitat was less than that compared to the mean overall horizontal distance covered per day across the entire trip. This suggests the individual was engaging in foraging activity, despite lower PCA rates (Fig 2). As such, the relationship between the number of PCAs and the horizontal distance covered per day was investigated separately for each of the oceanographic domains to assess whether PCA rates were consistent between these varying habitat types.

**Fig 3:**
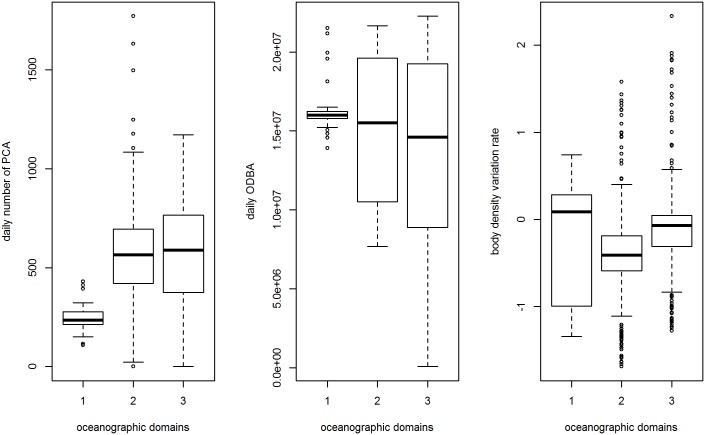
Daily numbers of prey catch attempts, daily energy expenditures (ODBA in *g*) and body density variation rates across all individuals per oceanographic domain.

The mean daily number of PCAs was lower in habitat 1 compared to the two other habitats (Fig 3). Conversely, mean daily body activity tended to be slightly higher, and generally exhibited much less variability, within habitat 1 compared to the two other habitats (Fig 3; although note a narrow variability may be due to the smaller number of individuals that visited this domain). Daily changes in body density also varied between oceanographic domains (Fig 3). In the second habitat (between the Sub-Antarctic Front and the Polar Front) body density decreased at a faster rate than that observed in the third habitat (south of the Polar Front), which reflected a decrease in the number of PCAs per day and a slight increase in swim effort (ODBA). In habitat 1, whilst the median rate of change in body density was positive and higher than the negative estimates from habitats 2 and 3, the inter-quartile range of these values is extensive and also encompasses negative rates of change similar to those observed in habitats 2 and 3.

**Fig 4:**
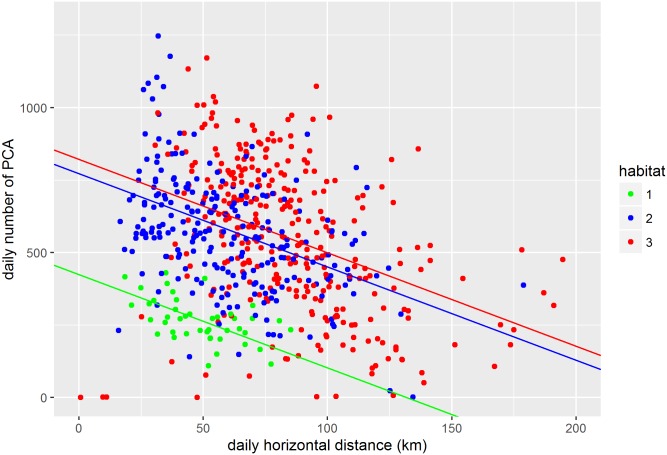
Plot of the linear mixed effects model comparing the number of PCAs in relation to the horizontal distance covered alongside the predominant oceanographic domain (habitat) occupied across each day with individual as a random effect (see S3 Fig).

A significant negative correlation was found between the number of PCAs and the horizontal distance travelled across a day, regardless of the oceanographic domain visited (Eq 3). There was an estimated decrease of 3.3 ±0.7 PCA for every kilometre covered horizontally per day (*parameter estimate =* -3.3 ±0.7, *p* < 0.001, see Fig 4 and S1 Table). Regarding oceanographic domain, the daily number of PCAs in both habitats 2 and 3 were significantly higher than those observed in habitat 1 (Habitat2: *parameter estimate =* 214.8 ±45.6, *p* < 0.001 and Habitat3: *parameter estimate =* 198.4 ±48.2, *p* < 0.001, with Habitat1 as the baseline for the categorical factor of oceanographic domain; see Fig 4 and S1 Table). The difference of daily mean PCAs between habitat 2 and habitat 3 was not significant (*parameter estimate =* -16.4 ±24.1, *p* = 0.49, with Habitat2 as the baseline for the categorical factor, oceanographic domain; Fig 4).

### Variation in SES body density

SES body density consistently decreased, in all tracked individuals, by around 0.9 ±0.8% across the duration of a trip (Fig 2). From the smoothed time series (Fig 2), a decrease in body density occurred approximately 10 to 20 days after the start of a trip. A steeper slope suggests a faster decrease in body density.

**Fig 5:**
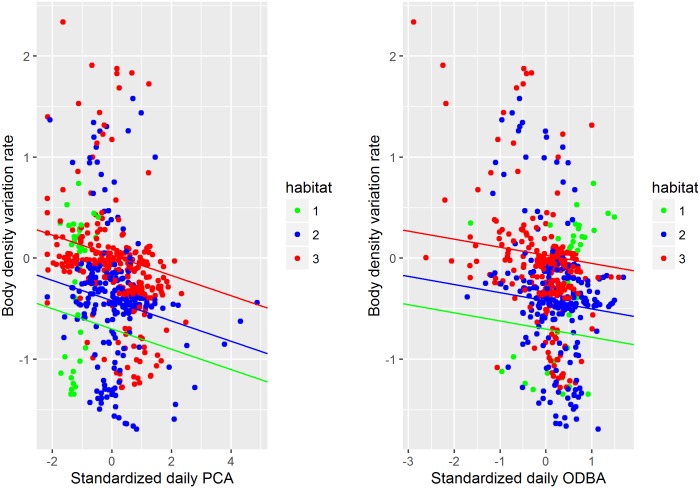
Plots of the linear mixed effects model comparing rate of body density change in relation to the number of PCAs (left box) and the body activity (right box) alongside the predominant oceanographic domain (habitat) occupied across each day with individual as a random effect (see Table 1).

Using LMM, we then assessed the relationship between body density variation rates and the number of prey capture attempts, the body activity, and the oceanographic domains, at a lag of 6 days (Eq 4). We found that a decrease in body density was positively related to both the number of PCAs and body movement 6 days before (Table 1 and Fig 5). However, the number of PCAs and body movement (ODBA) were not correlated (cor = -0.4). As such, it appears an increase in PCA induces a decrease in SES body density. Similarly an increase in body movement leads to a decrease in body density at a lag of 6 days. The highest difference of body density variation rate was found between habitat 3 and habitat 1 (*Habitat3 Estimate =* 0.73 ±0.15, *p* < 0.001), suggesting that, for a given PCA and energy expenditure, the rate of change in body density is significantly higher in habitat 3 than in habitat 1. Similarly, the rate of change in body density was significantly higher in habitat 2 than in habitat 1 (*Habitat2 Estimate =* 0.28±0.13, *p*<0.001, see Table 1 and Fig 5), but significantly lower than in habitat 3 (with Habitat2 as a baseline for the categorical factor, *Habitat3 Estimate =* 0.46±0.07, *p*<0.001, see Fig 5). In other words, since a negative rate of change in body density indicates a decrease in body density (Eq 2), the body densities of SESs foraging north of the sub-Antarctic front (habitat 1) decreased at a faster rate given a set level of body movement and a number of PCAs, while the body densities of SESs foraging south of the polar front decreased at a slower rate (habitat 3, see Table 1 and Fig 5). We assessed individual variability in body density (*i*.*e*. the variability within the oceanographic domains for a given number of PCA and a given body activity) from the random component of the model (which included individual as a random effect). We observed a standard deviation of 0.21, meaning that whilst there is inter-individual variability, it is less than the differences between oceanographic domains (Table 1 and Fig 5).

**Table 1:** Results from linear mixed effects models investigating the rate of body density change in relation to the number of PCAs, energy expenditure and the predominant habitat occupied. For the categorical variable, the baseline is set at habitat 1, *i*.*e*. values for habitats 2 and 3 in the table are the differences between their estimated coefficients and the ones for habitat 1. Significant variables at 0.05 are highlighted in bold. A random effect of individual was included on the intercept: std = 0.21. The difference in rate of body density change between habitat 2 and 3 is: Estimate = 0.46, std = 0.07, t = 6.1, p<0.001 (with habitat 2 set as the baseline).

LAG 6 days	Value	Std error	T-value	P-value
Intercept	-0.70	0.14	-4.91	<0.001
Daily number of PCA	-0.10	0.03	-3.14	0.002
Daily ODBA	-0.08	0.03	-2.993	0.003
Habitat2	0.28	0.13	2.11	0.036
Habitat3	0.73	0.15	5.05	<0.001

## Discussion

We describe variation in the foraging behaviour of SES females over their trip and between oceanographic domains, which possibly reflects changes in foraging efficiency. We show that variation in body density is related not only to the number of previous prey catch attempts, but also foraging habitat type. We suggest these results reflect differences in the quality and/or quantity of prey encountered between oceanographic domains.

### Horizontal distance as a foraging predictor

Throughout SES trips, different trip phases were identified based upon horizontal travel distances and variation in the number of prey catch attempts. This finding supports the previous use of area-restricted search behaviours [32,34,35], suggesting that a reduced horizontal speed does indeed coincide with increased foraging activity. Faster and more directed travels were mainly observed at the beginning and end of a foraging trip (*i*.*e*. when SES females are leaving or travelling back to their colony [33] and individuals perform mainly travelling dives [32]). During the middle part of a foraging trip, SESs covered smaller distances and increased feeding activity, suggesting more intensive foraging activity [32,35]. Variation in dives metrics, rather than variation in indices of horizontal travel, are known to explain most of the variation in the number of PCAs [25].

### Variation in body density

The body densities of all female SESs decreased during trips, suggesting a replenishment of lipid reserves. However, this improvement in body condition (*i*.*e*. decrease of body density) is not linear, since SESs increase their body density only slightly during the first few days after their departure. We assumed that before SESs attempt to replenish depleted lipid reserves, individuals either burn their last fat reserves or allocate net energy gains to muscle (denser than fat) synthesis at the very beginning of a foraging trip. The latter of these scenarios is to restore depleted protein stores which are low following a prior month of fasting that accompanies a period of inactivity. However, whilst both these scenarios would explain an increase in body density [9,19,21,23] at the beginning of a SES’s trip, low PCA rates at both the beginning and end of a foraging trip, when body density tends to level off or increase, possibly suggests that females favour travelling activity over foraging activity and are not catching enough prey to be able maintain/improve their condition. Therefore, this increase in body density is most likely explained by a further depletion of their fat store rather than the allocation of new net energy gain to muscle synthesis.

As expected, an improvement in SES body condition (*i*.*e*. an increase of lipid proportion) was positively related to the number of PCAs, such that the higher the number of PCAs the faster a SES’s body condition improved. Body movement was also positively related to improved body condition at a 6-day lag (*i*.*e*. when seals increase their swim activity while foraging, they improve their body condition at a faster rate). While we expected SESs that exhibited increased body activity for a given number of PCAs to improve their body condition less rapidly, we found the opposite. This result suggests that body movement is possibly indicative of increased foraging activity, such that SESs exhibiting higher levels of body activity during foraging dives are possibly catching larger and/or richer prey.

### Does foraging efficiency differ between oceanographic domains?

Our sampling covered three oceanographic domains, although note that only two individuals occurred in the region north of the sub-Antarctic front (habitat1). However, using individuals as random effect strengthened our models, allowing us to compare oceanographic domains. Although recorded trips were not always complete, all SESs were tracked in excess of 30 days, which we consider long enough to observe variations of body condition. Indeed, Richard et al. [19] showed that a decrease of body density generally occurs around 15 days into a trip.

This study highlights differences in the rate of change in body density between varying habitat types, and in tandem with findings from previous studies [24,36,37], suggests that the oceanographic domain visited by a SES has an effect on its rate of body density variation. Indeed, Hindell et al. [35] revealed the importance of foraging in shelf waters for different SES populations, which confirms previous assumptions that these types of foraging habitats aid fast lipid replenishment [36,37]. However, Kerguelen SESs forage preferentially in the Southern Indian Ocean region, spending very little time in high quality Antarctic shelf waters, probably due to the long journey necessary to access these areas [35]. Nevertheless, SESs from Kerguelen may forage within the continental shelf waters of their island, although this is generally only observed in males and less so in females [38]. The females of this study may forage in the deep ocean to avoid intra-specific competition with males [38,39] or to avoid a higher predation risk by killer whales (*Orcinus orca*) or sleeper sharks (*Somniosus antarcticus*) [40]. Considering the foraging habitats of Kerguelen SES females, for a similar body activity and a similar number of PCAs, seals foraging north of the sub-Antarctic front (habitat 1) would decrease their body density at a faster rate than seals foraging south of the sub-Antarctic front (habitat 2) and the polar front (habitat 3) where seals have the slowest rate of change in body density. However, when foraging north of the sub-Antarctic front (habitat 1) SES females performed half the number of PCA per day (266±75 PCAs.d^-1^) compared to when foraging south of the sub-Antarctic front (577±222 PCAs.d^-1^, habitat 2) and south of the polar front (545±255 PCAs.d^-1^, habitat 3). We thus assumed that depending on the foraging habitat, SESs are feeding on prey of different sizes and/or qualities. It may be that female SES are more successful, needing fewer prey catch attempts and/or consuming larger or richer prey, when foraging north of the sub-Antarctic front than in other habitats. Indeed, the largest myctophid species, such as *Gymnoscopelus nicholsi* (~30g), *Gymnoscopelus piabilis* (~30g) and *Gymnoscopelus bolini* (~200g, C. A. Bost & Y. Cherel pers. comm.), are thought to be more abundant north of the Subantarctic front [13,41,42], whilst smaller species, such as *Electrona carlsbergi* (~9g) and *Electrona antarctica*, (~9g, C. A. Bost & Y. Cherel pers. comm.) dominate the myctophid communities south of the sub-Antarctic front [13,41,42]. In addition, deeper SES dive depths suggest these resources are vertically less accessible in the domain north of the sub-Antarctic front [13,14,43] resulting in an increase in locomotion costs and dive transit durations [23]. Indeed, although overall differences in locomotion costs between habitats were not significant (probably due to the small sample size of SESs visiting habitat 1), but seals foraging north of the sub-Antarctic front tended to exhibit the largest ODBAs. In accordance with optimal foraging/diving theory [44], our results indicate that these increased foraging costs north of the sub-Antarctic front, are related to the diving depths of SESs [19,45], and likely compensated by targeting larger/better quality prey. This allows a greater energetic intake per unit of time spent foraging at the bottom of the dive.

This study provides important evidence that an increase in lipid proportion in post-breeding females SESs is possibly driven by variation in both the quantity and quality of prey ingested. However, to completely resolve this, and elucidate the mechanisms driving differences in foraging efficiency between the oceanographic domains, further investigations involving additional SES foraging activity from north of the sub-Antarctic front would be required, alongside indications of prey fields.

## Supporting Information

S1 Fig Examples of smoothed body density across a trip of one SES several with 4 different smoothing parameters. Smoothing parameters were fitted and the best chosen based on visual observation.
(PDF)

S2 Fig Daily horizontal distances, the number of prey catch attempts and the body densities across each recorded trip for the 10 southern elephant seals. Colour codes of each value relate to the major oceanographic domains visited during the day: habitat 1 in green, habitat 2 in blue and habitat 3 in red.
(PDF)

S3 Fig Daily numbers of PCA for each individual and per oceanographic domains. Colour codes of each value relate to the major oceanographic domains visited during the day: habitat 1 in green, habitat 2 in blue and habitat 3 in red.
(PDF)

S1 File Data with the number of PCAs, the horizontal distance covered and the predominant oceanographic domain occupied across each day (Eq 3). (CSV)

S2 File Data with body density changes, number of PCAs, body activity and predominant oceanographic domain occupied across each day (Eq 4). (CSV)

S1 Table Result from the linear mixed-effects model comparing the number of PCAs in relation to the horizontal distance covered alongside the predominant oceanographic domain (habitat) occupied across each day, with individual as random effect. For the categorical variable, the baseline level is set for habitat 1, *i*.*e*. values for habitats 2 and 3 in the table are the differences between their estimated coefficient and the one of the habitat 1. Significant variables at 0.05 are highlighted in bold. Random effects Std dev of intercept = 224. The difference between habitat 2 and 3 is: Estimate = -16.4, std = 24.1, t = -0.7, p = 0.49 (ns).
(PDF)
