# Supplementary figures and images for: Southern Elephant Seals Replenish Their Lipid Reserves at Different Rates According to Foraging Habitat
Source: PLoS One. 2016 Nov 30;11(11):e0166747. doi: 10.1371/journal.pone.0166747 (PMC5130208; doi:10.1371/journal.pone.0166747)

$p=0.01$

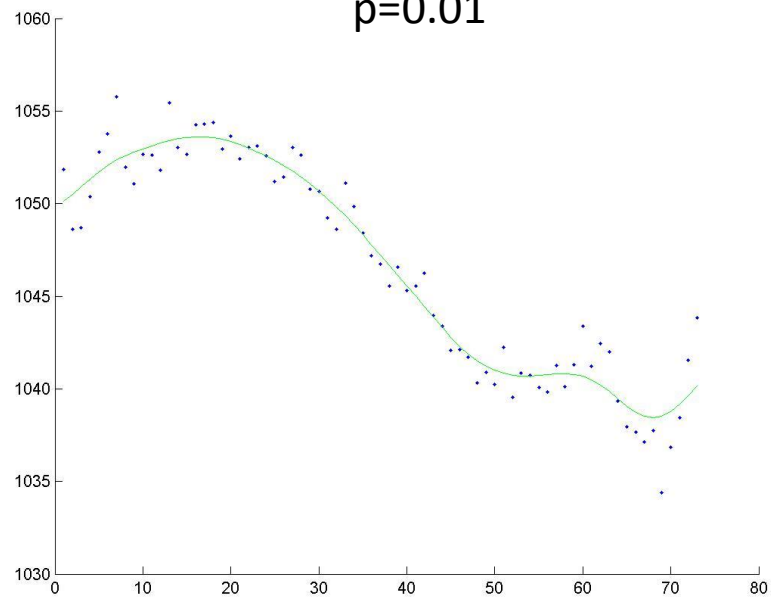

$p=0.05$

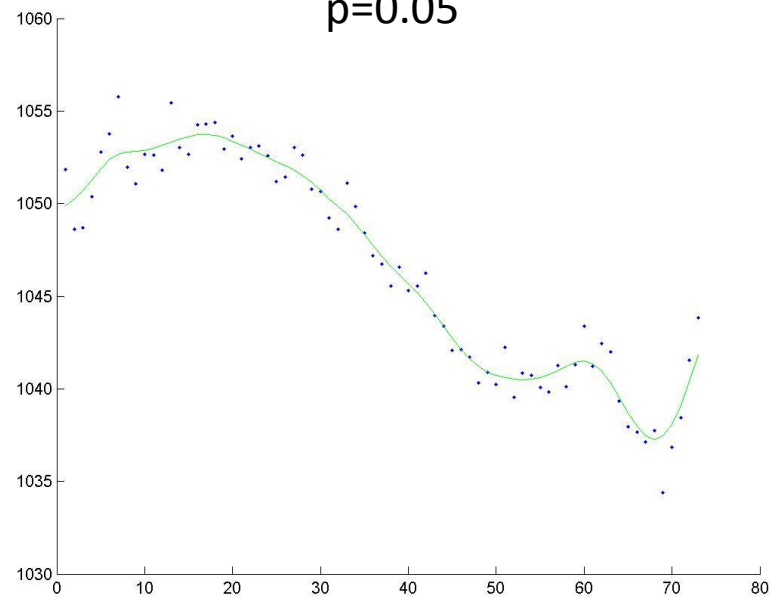

$p=0.1$

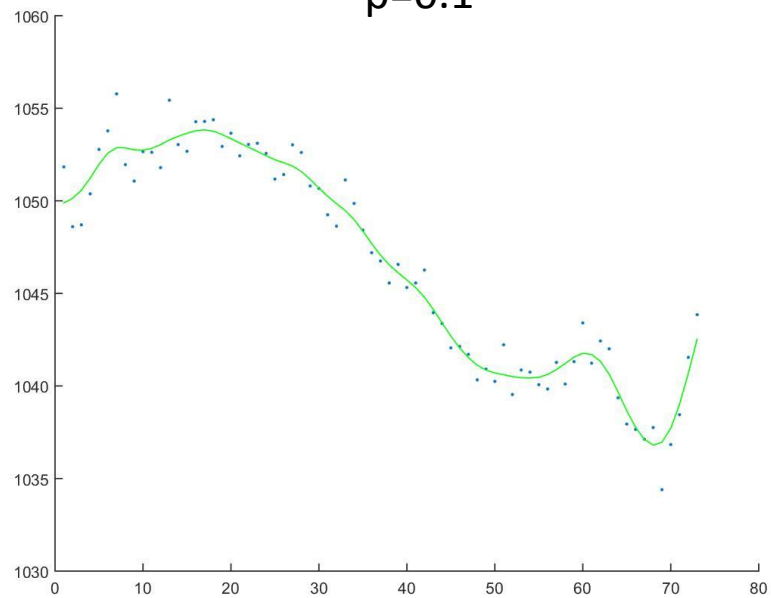

$p=0.5$

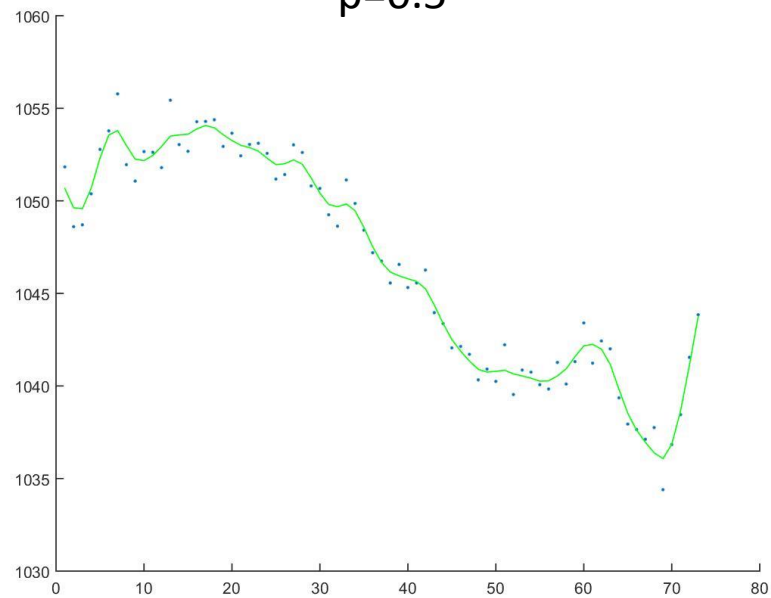

Supplement: S1 Fig — Smoothing parameters were fitted and the best chosen based on visual observation. (PDF) [file pone.0166747.s001.pdf]

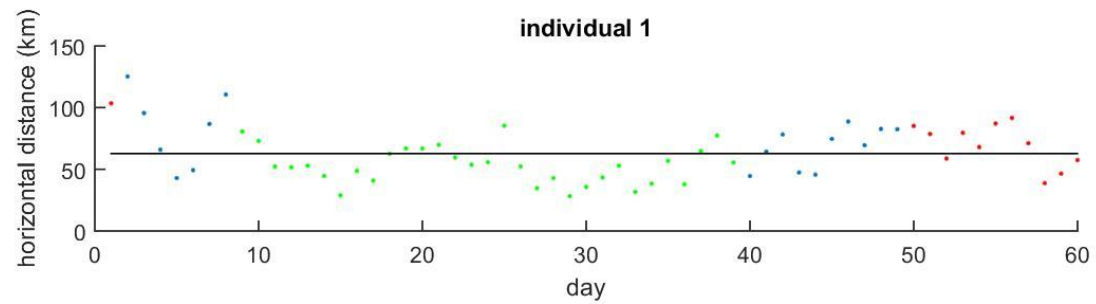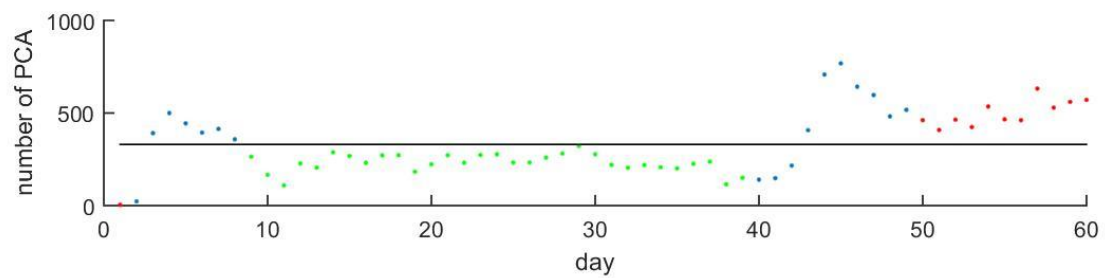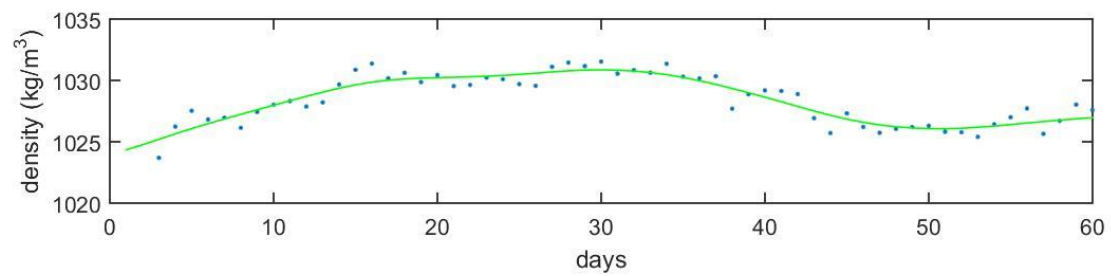

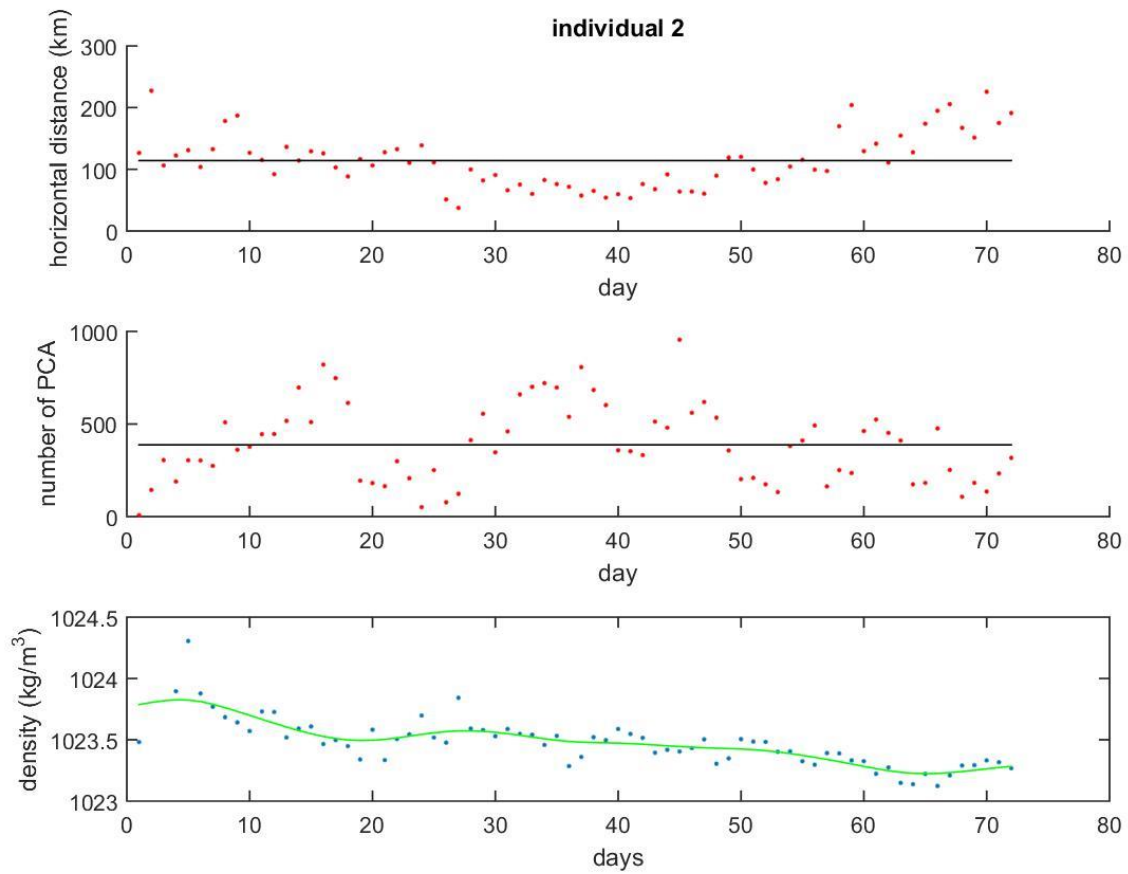

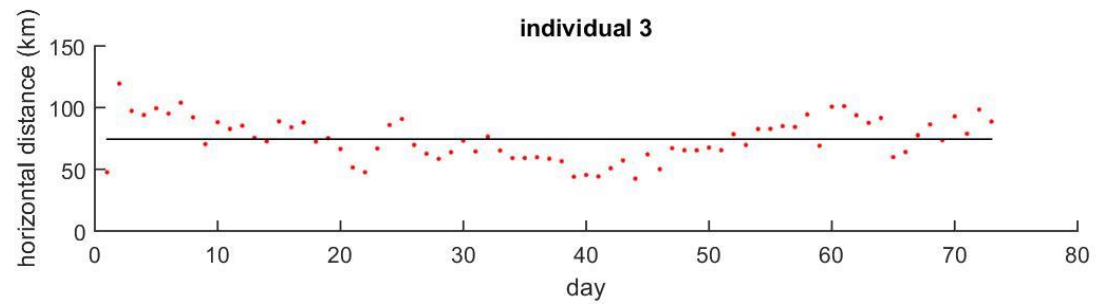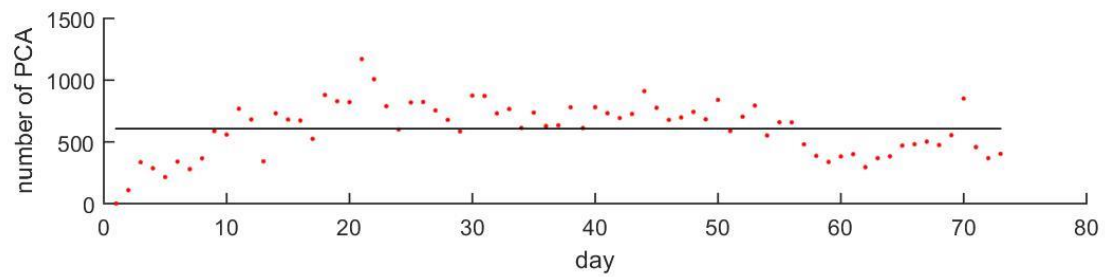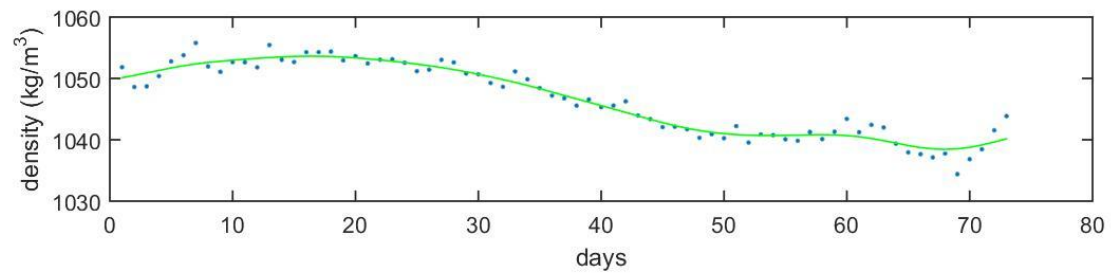

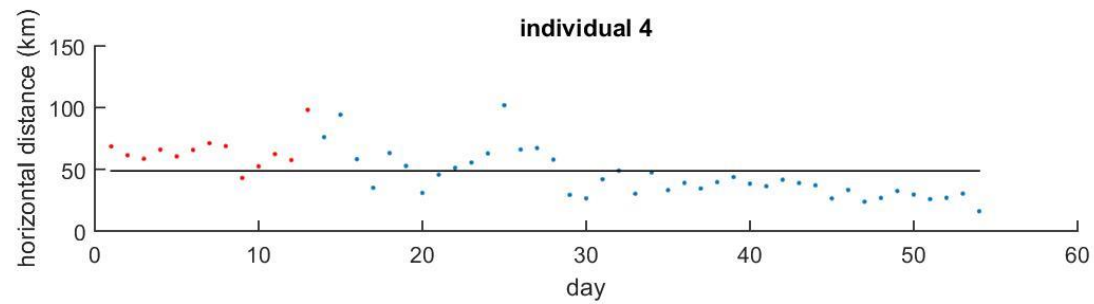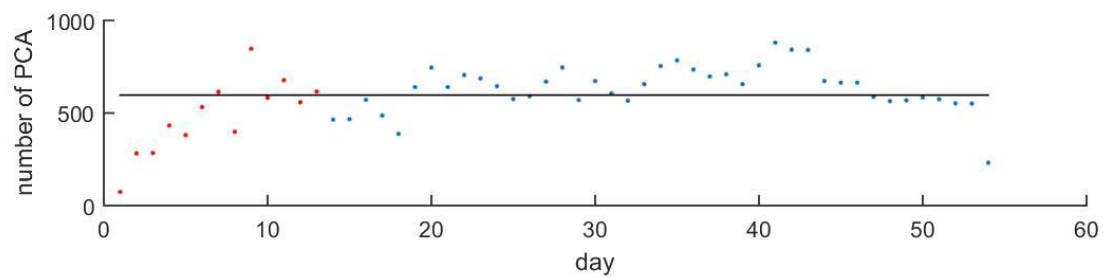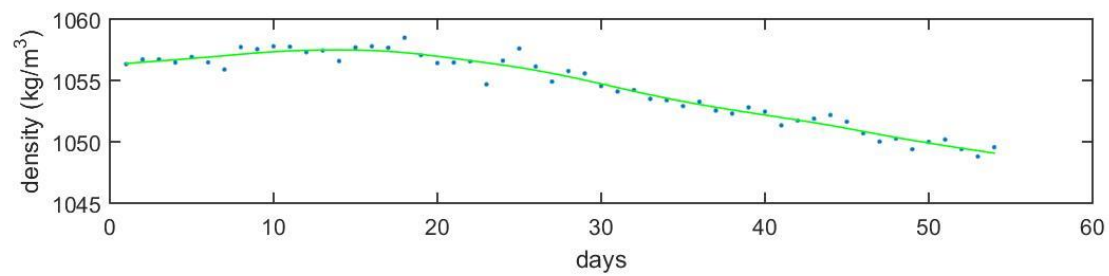

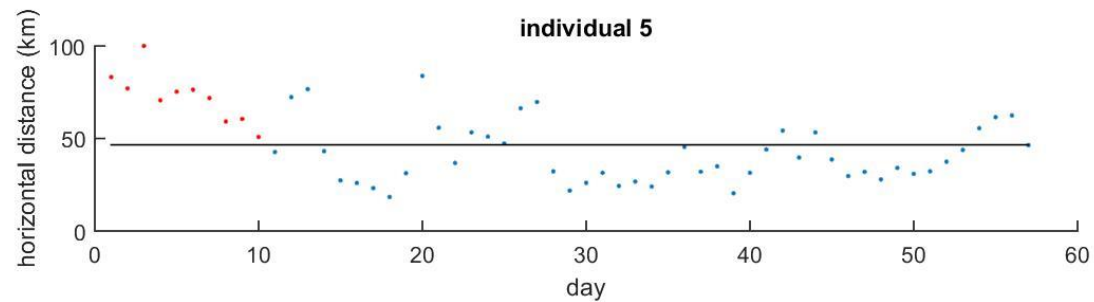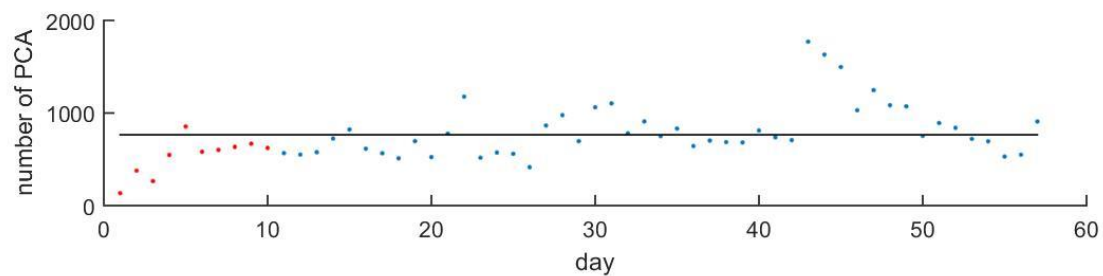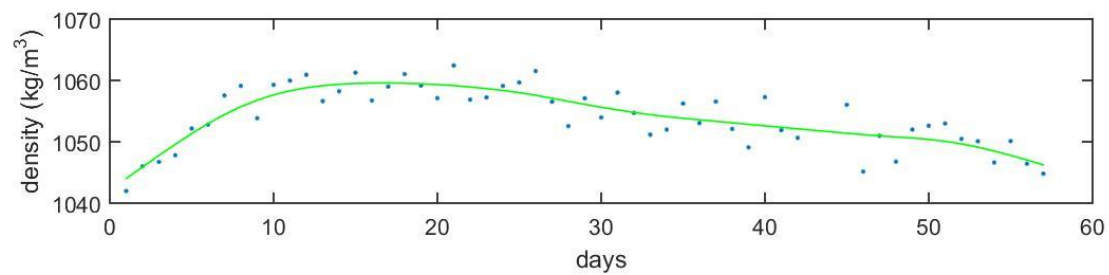

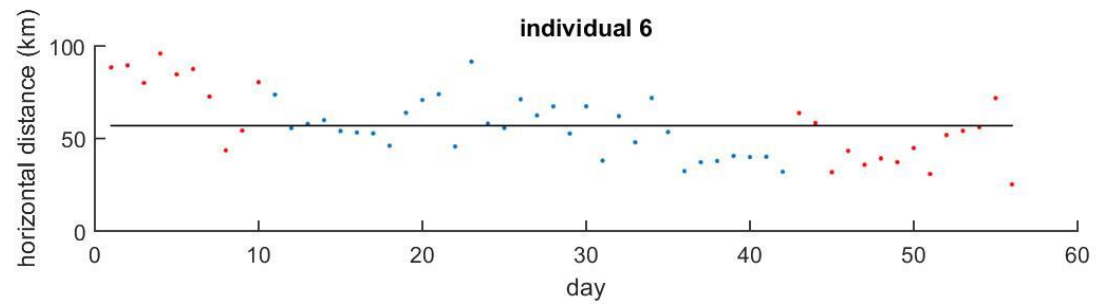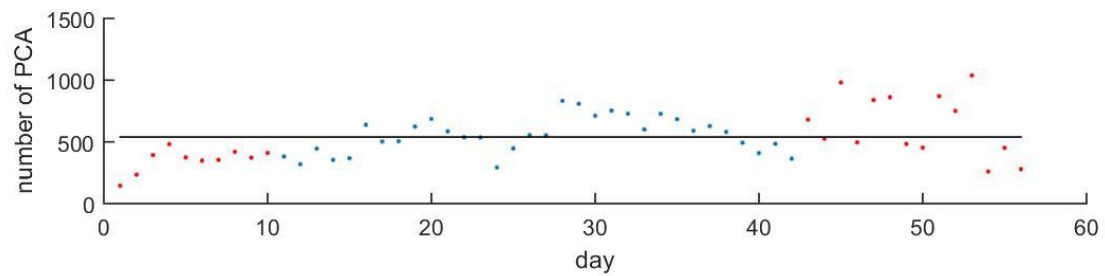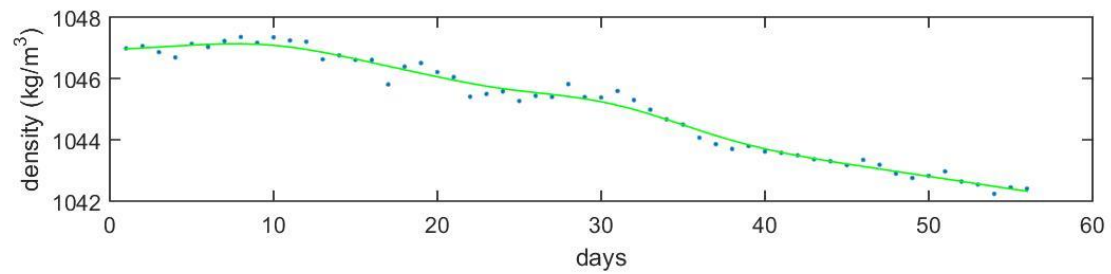

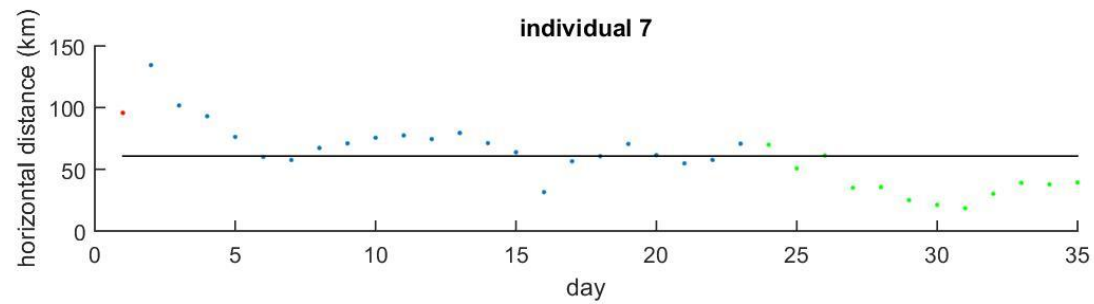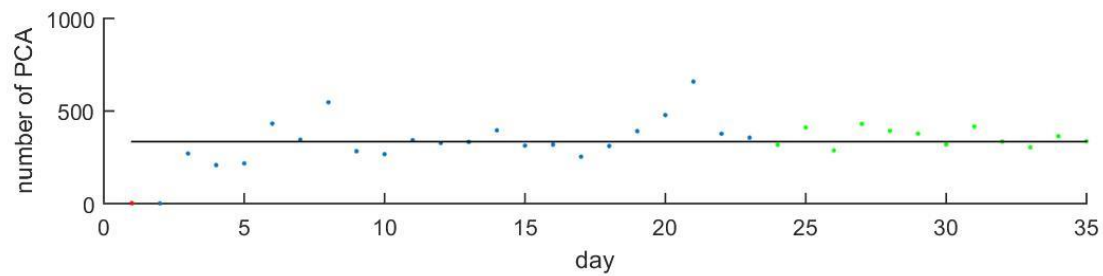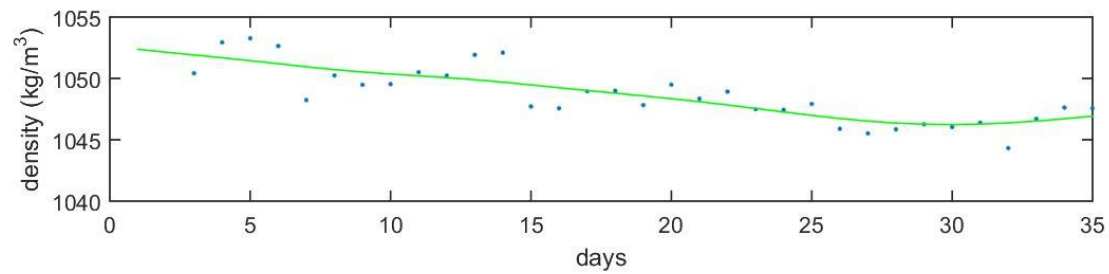

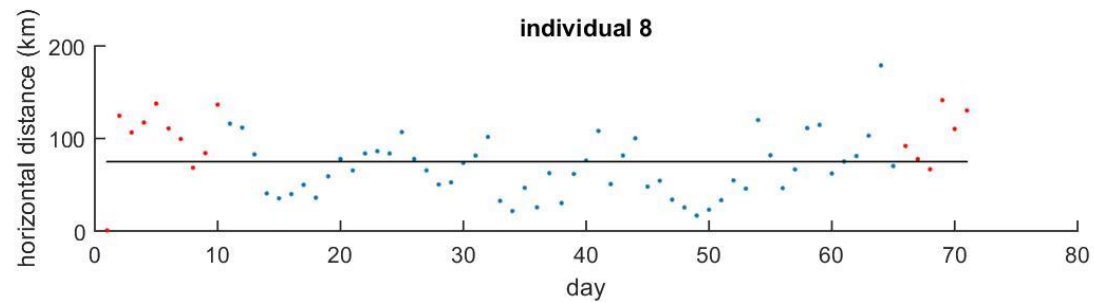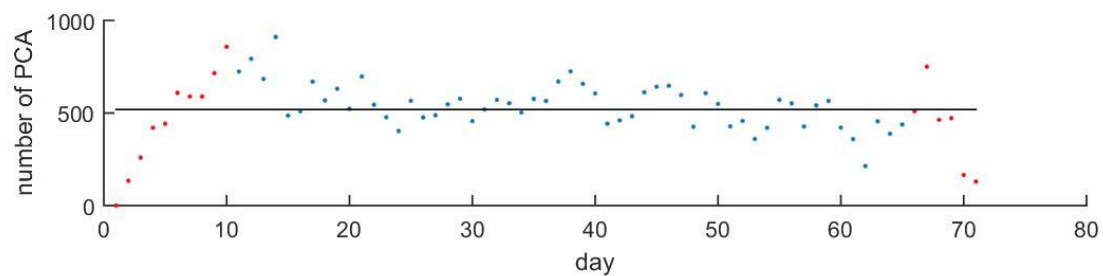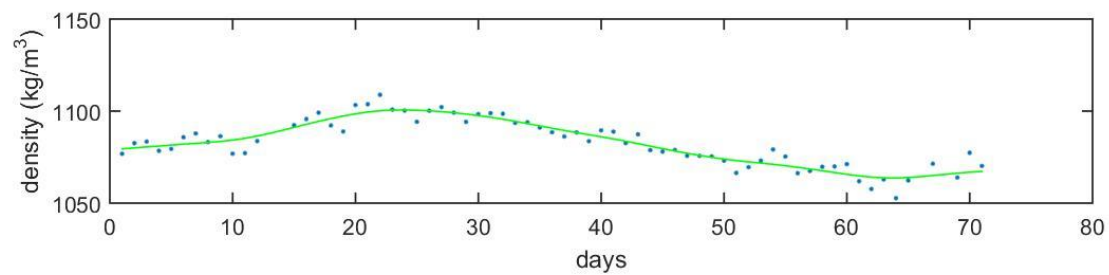

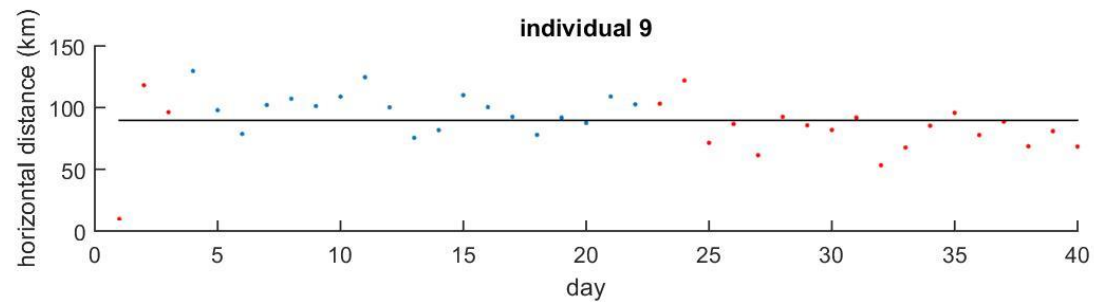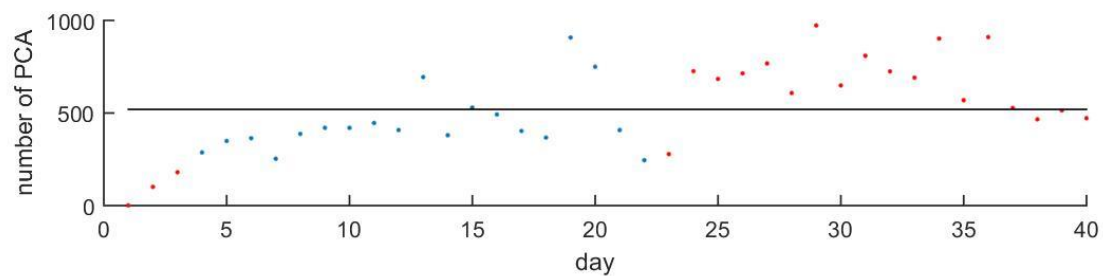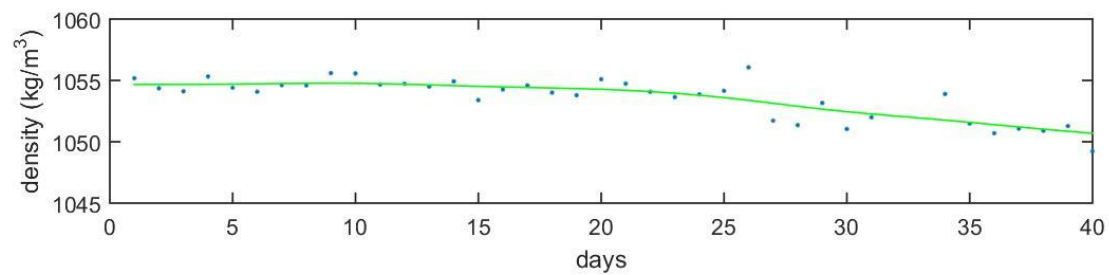

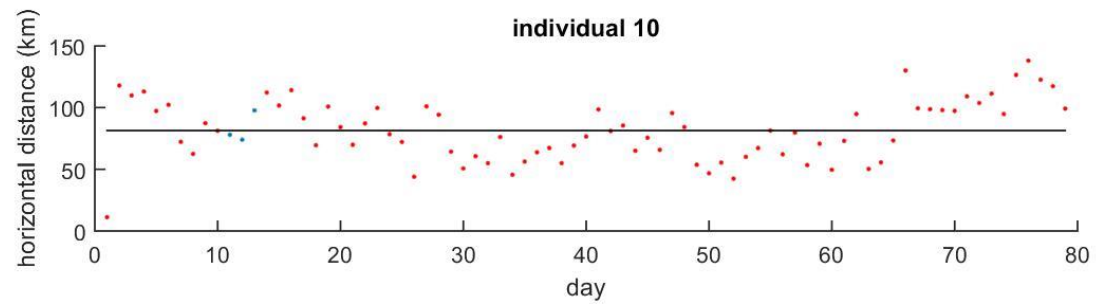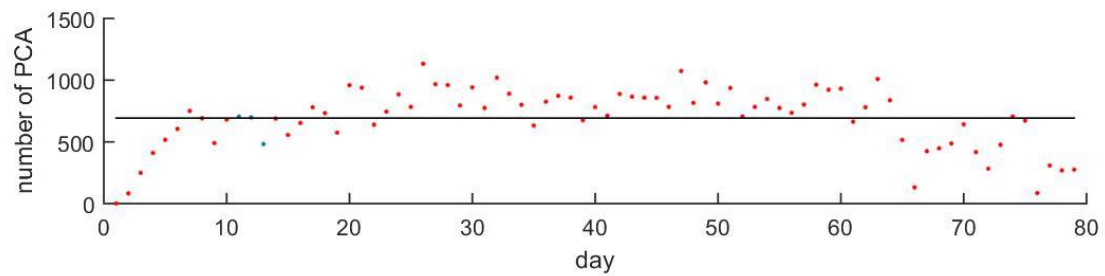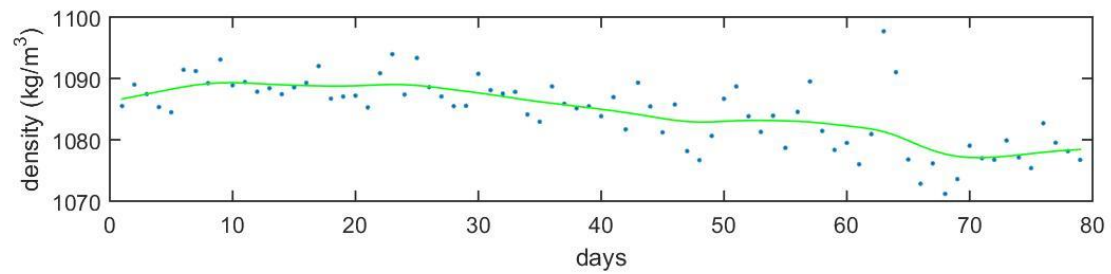

Supplement: S2 Fig — Colour codes of each value relate to the major oceanographic domains visited during the day: habitat 1 in green, habitat 2 in blue and habitat 3 in red. (PDF) [file pone.0166747.s002.pdf]

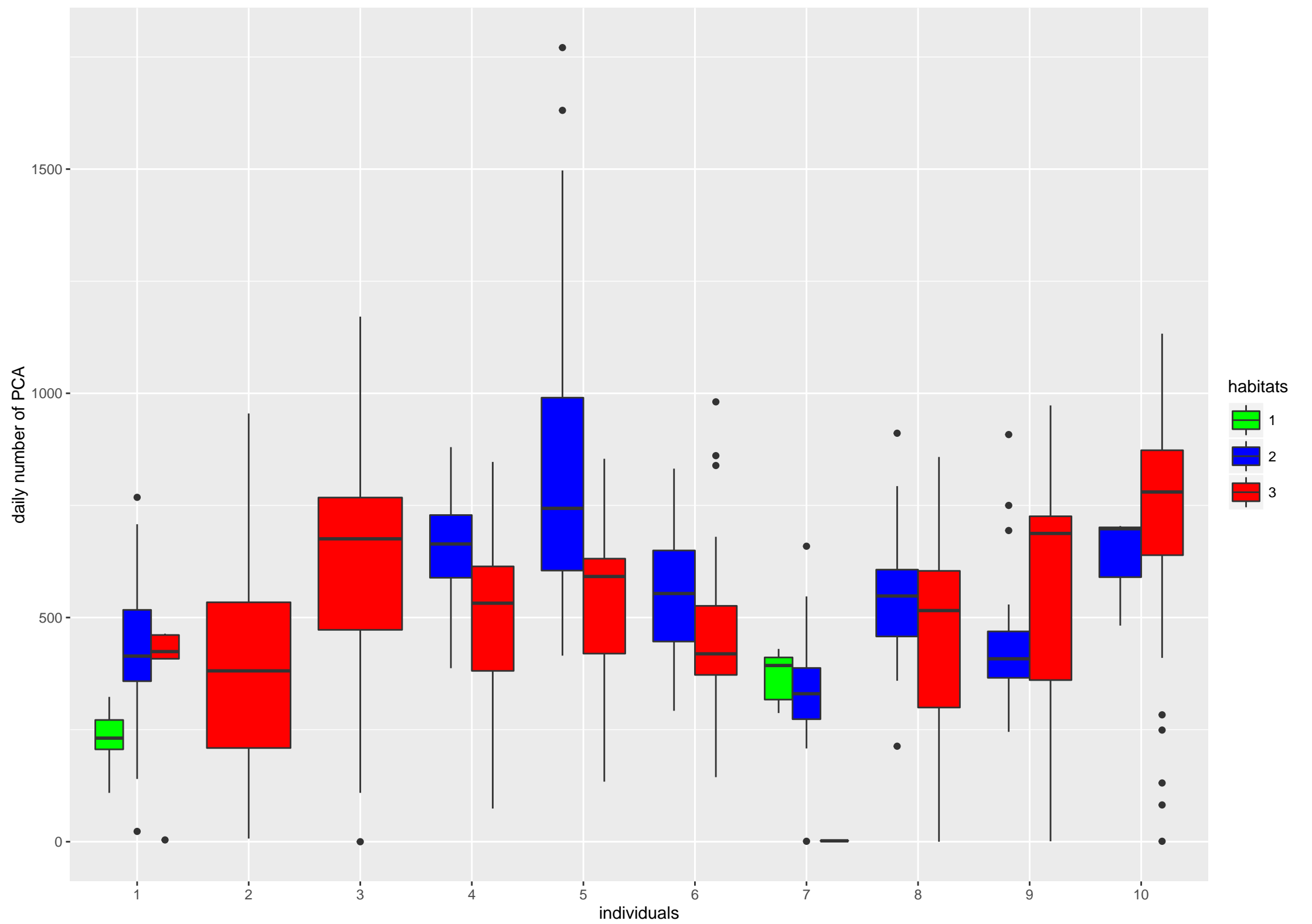

Supplement: S3 Fig — Colour codes of each value relate to the major oceanographic domains visited during the day: habitat 1 in green, habitat 2 in blue and habitat 3 in red. (PDF) [file pone.0166747.s003.pdf]
