# Supplementary material for: Southern Elephant Seals Replenish Their Lipid Reserves at Different Rates According to Foraging Habitat
Source: PLoS One. 2016 Nov 30;11(11):e0166747. doi: 10.1371/journal.pone.0166747 (PMC5130208; doi:10.1371/journal.pone.0166747)
Supplement: S1 Table — For the categorical variable, the baseline level is set for habitat 1, i.e. values for habitats 2 and 3 in the table are the differences between their estimated coefficient and the one of the habitat 1. Significant variables at 0.05 are highlighted in bold. Random effects Std dev of intercept = 224. The difference between habitat 2 and 3 is: Estimate = -16.4, std = 24.1, t = -0.7, p = 0.49 (ns). (PDF) [file pone.0166747.s006.pdf]

	Value	Std error	T-value	P-value
Intercept	567.3	82.8	6.9	<0.001
Distance	-3.3	0.7	-4.5	<0.001
Habitat2	214.8	45.6	4.7	<0.001
Habitat3	198.4	48.2	4.1	<0.001

S4 Table. Results from the linear mixed-effects model comparing the number of PCAs in relation to the horizontal distance covered alongside the predominant oceanographic domain (habitat) occupied across each day, with individual as a random effect.

For the categorical variable, the baseline level is set for habitat 1, *i.e.* values for habitats 2 and 3 in the table are the differences between their estimated coefficient and the one of the habitat 1. Significant variables at 0.05 are highlighted in bold. Random effects Std dev of intercept = 224.

The difference between habitat 2 and 3 is: Estimate= -16.4, std= 24.1, t=-0.7, p=0.49 (ns).
